# U-Shaped and Surface Functionalized Polymer Optical Fiber Probe for Glucose Detection

**DOI:** 10.3390/s18010034

**Published:** 2017-12-25

**Authors:** Mikel Azkune, Leire Ruiz-Rubio, Gotzon Aldabaldetreku, Eneko Arrospide, Leyre Pérez-Álvarez, Iñaki Bikandi, Joseba Zubia, Jose Luis Vilas-Vilela

**Affiliations:** 1 Department of Communications Engineering, University of the Basque Country (UPV/EHU), Engineering School of Bilbao, Plaza Ingeniero Torres Quevedo, 1, E-48013 Bilbao, Spain; gotzon.aldabaldetreku@ehu.eus (G.A.); inaki.bikandi@ehu.eus (I.B.); joseba.zubia@ehu.eus (J.Z.); 2 Macromolecular Chemistry Research Group (LABQUIMAC), Department of Physical Chemistry, Faculty of Science and Technology, University of the Basque Country (UPV/EHU), Barrio Sarriena s/n, E-48940 Leioa, Spain; leire.ruiz@ehu.eus (L.R.-R.); leyre.perez@ehu.eus (L.P.-Á.); joseluis.vilas@ehu.eus (J.L.V.-V.); 3 Department of Applied Mathematics, University of the Basque Country (UPV/EHU), Torres Quevedo 1, E-48013 Bilbao, Spain; eneko.arrospide@ehu.eus

**Keywords:** polymer optical fibers, absorption evanescent wave sensing, bio-chemical sensing, surface functionalization

## Abstract

In this work we show an optical fiber evanescent wave absorption probe for glucose detection in different physiological media. High selectivity is achieved by functionalizing the surface of an only-core poly(methyl methacrylate) (PMMA) polymer optical fiber with phenilboronic groups, and enhanced sensitivity by using a U-shaped geometry. Employing a supercontinuum light source and a high-resolution spectrometer, absorption measurements are performed in the broadband visible light spectrum. Experimental results suggest the feasibility of such a fiber probe as a low-cost and selective glucose detector.

## 1. Introduction

The attention of the scientific community has been focused on low-cost, sensitive, and specific alternatives for the detection and analysis in low concentration solutions of different substances due to the demand in many fields such as clinical diagnosis, drug discovery, food safety, etc., [1]. In this sense, glucose sensing and monitoring is regarded as a critical indicator, so that fabricating reliable solutions would result in a step forward [2]. Biosensors interact with a target molecule, cell, or any other biological element and produce a calibrated signal that is used as a parameter transducer [3]. The combination of optical fiber devices with chemically sensitive coatings offers a universal platform for the development of highly sensitive and selective sensors [4]. 

The use of polymer optical fibers (POF) fulfils perfectly the requirements of biosensors and provides many advantages in comparison to other types of sensors, mainly owing to the high interaction lengths with the sample and the transmitted light as well as the simplicity of the sensors [5]. POFs are usually made up of poly(methyl methacrylate) (PMMA), a cheap and transparent polymer. Sensors based on POFs are in general low-cost and easy to use compared with other time-consuming traditional methods [6,7,8]. For instance, fiber-optic evanescent wave (FOEW) absorption sensors are widely used, and they rely on detecting a change on the transmitted spectrum due to the coupling of the light to the surrounding medium. This phenomenon is enhanced either by tapering the fiber or by bending the fiber in a U-shape [9]. In most cases, these FOEW sensors depend on indirect measurements to obtain the concentration of glucose, such as measuring the change in the refractive index of the solution induced by the presence of glucose. In contrast, the sensor presented in this work selectively attaches the glucose and releases a reporter, making the sensor media-independent and straightforward.

In order to create a sensor with high selectivity that is capable of attaching a specific target separated from other undesired substances, the outer surface of the POF must be specifically functionalized. For that purpose, the PMMA POF probe surface is functionalized with phenylboronic acid (PBA) groups. The glucose sensing behavior of these groups is based on the interaction between boronic acid and glucose, forming a cyclic boronate ester.

The measurement principle relies on the release of an optical reporter called Alizarin Red S (ARS), which is bonded to the fiber by a reversible interaction with boronic acid groups. The sensing system is composed by a 500-μm uncladded POF bent in a U-shape with a 2.5 mm diameter. By immersing the sensitive area of the probe in a solution containing glucose, the ARS is released and, therefore, a change in the absorption spectra is observed. Employing an ad hoc designed experimental setup, these absorption spectra are measured and recorded. The obtained results demonstrate that our sensor is able to measure glucose in many different media at biological pH levels.

## 2. Working Principle of the Sensor

Light is launched from one end of the fiber and the spectrum of the transmitted light is measured at the other end. This transmission spectrum depends on the absorption of the evanescent field penetrating into the fluid that acts as a cladding. On the one hand, the U-shaped fiber probe gives the proper sensitivity and, on the other hand, the surface functionalization ensures the required selectivity. The principles of both fields will be explained below.

### 2.1. Evanescent Wave Sensing

Evanescent waves are well documented in the bibliography [9,10] and have been used in many biosensors [4,6,11,12,13,14]. They are confined between the core and the cladding of the optical fiber and are associated to a loss or leakage of the transmitted signal.

**Figure 1:**
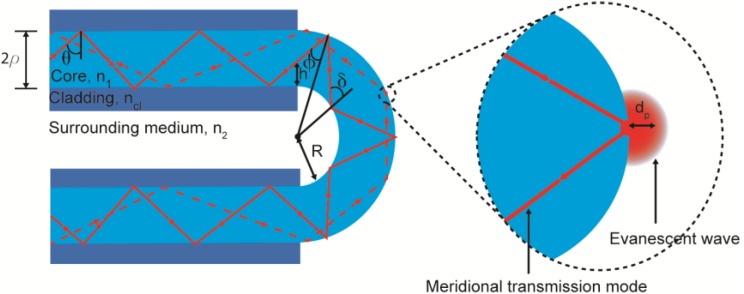
Geometry of the U-shaped sensing region and the representation of a meridional transmission mode (**left**); illustration of the penetration depth (**right**).

During light transmission through an optical fiber, the evanescent wave decays exponentially with the distance from the core-cladding interface until its intensity is negligibly small [3]; this parameter is defined as the penetration depth (*d_P_*) (Figure 1). This depth defines the distance in which the molecules may have a discernible effect in the evanescent wave [15]:

(1) $$d_{P}=\frac{\lambda }{2\pi n_{1}{\left(\cos^{2}\theta_{c}-\cos^{2}\theta \sin^{2}\theta_{\phi }\right)}^{1/2}}$$

where *λ* is the vacuum wavelength of the light launched to the fiber, *n*_1_ is the refractive index of the core, *θ_c_* is the critical angle in the sensing region with respect to the normal to the core-cladding interface, *θ* is the angle of the wave with the normal to the core-cladding interface, and *θ_φ_* is the skewness angle [15] (which is π/2 for a meridional transmission mode).

The *d_P_* of an evanescent wave is very small in a straight fiber, but it can be notably increased by bending the fiber. Thus, using a U-shaped bending enhances the sensitivity of the fiber probe. Furthermore, the analysis of the skewness can be split depending on whether the light interacts with the outer or the inner surface. In the former case, the skewness angle changes from:

(2) $$\phi_{1}=\sin^{-1}\left[\left(\frac{R+h}{R+2\rho }\right)\frac{n_{cl}}{n_{1}}\right],$$

to:

(3) $$\phi_{2}=\sin^{-1}\left[\frac{R+h}{R+2\rho }\right],$$

where *R* is the bending radius of the probe, *ρ* is the radius of the fiber core, and *h* is the height at the entrance of the bent region from the inner core-cladding interface. At the inner surface, the angle goes from:

(4) $$\delta_{1}=\sin^{-1}\left[\left(\frac{R+h}{R}\right)\frac{n_{cl}}{n_{1}}\right],$$

to δ_2_ = π/2. Using these equations, it can be proved that *d_P_* is much higher for U-shaped bent fibers than for straight fibers (*R* = ∞) [16]. Moreover, the absorbance is higher for smaller diameters and for lower numerical apertures [12]. 

### 2.2. Phenil-Boronic Acid Diol Interaction

**Figure 2:**
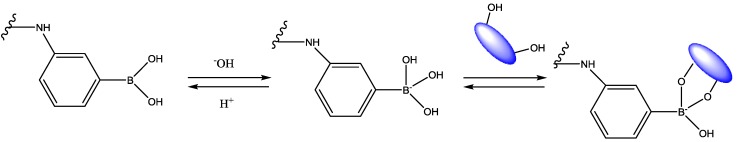
Simplified 3-aminophenylboronic acid (APBA) equilibrium in the presence of glucose.

Boronic acids bind compounds containing diol moieties with high affinity, forming reversible boronate esters [17]. Consequently, boronic acid compounds have widely been used for the synthesis of artificial receptors for sugars with great success [18]. The scheme in Figure 2 depicts a substrate containing 3-aminophenylboronic acid (APBA), which is a synthetic molecule capable of forming reversible boronates with 1,2-diol, 1,3-diol, or multi-hydroxyl groups including glucose [19]. Boronic acid-diol binding reactions are highly pH-dependent [20], and pH values above the pKa of the boronic acid are required, so that this study makes use of buffers usually employed for biological applications (Phosphate Buffered Saline System (PBS) and Tris base (TRIS)) to control the pH of the media [21]. However, it can be difficult to monitor the binding without using any fluophore.

**Figure 3:**
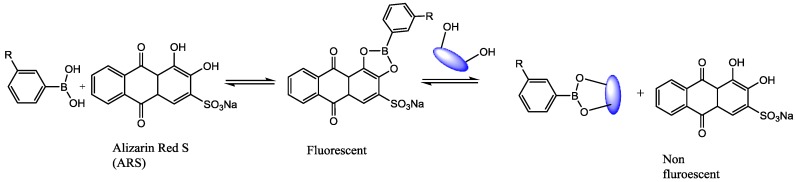
Competitive binding of phenylboronic acid (PBA) with Alizarin Red S (ARS) and glucose.

ARS has been used as a reagent for the fluorimetric determination of boronic acid concentrations. Free ARS is an organic dye with a very poor fluorescence. However, when ARS interacts with boronic acid groups, the active protons responsible for the fluorescence quenching are removed (Figure 3), leading to a dramatic increase in the fluorescence intensity of ARS [22,23]. In this work, ARS was used as an optical reporter. In the presence of glucose, the ARS is displaced from the boronic acid complex so the change in the absorption of the reporter allows the glucose detection by UV-Vis spectroscopy.

In summary, a PMMA fiber containing PBA was developed [19]. Firstly, the fiber surface was functionalized with PBA groups and bound with ARS. Secondly, the fiber was immersed in a solution containing 1,2-diol analytes. The competitive nature of boronic acid/diols complexes will favor the ARS-PBA bond breaking and glucose-boronic acid bond formation. The careful design of the experiment ensures that only the interaction of glucose and boronic acid can cause the ARS-boronic acid disruption. 

## 3. Experimental

APBA, ARS, *N*-(3-Dimethylaminopropyl)-*N*′-ethylcarbodiimide hydrochloride (EDC), *N*-Hydroxysuccinimide (NHS), glucose, PBS, and TRIS were purchased from Sigma Aldrich (St. Louis, MO, USA). Sulfuric Acid (96% purity), Hydrochloric Acid (HCl), ethanol, and isopropanol were purchased from Panreac (Spain), and were used without further purification.

PMMA of optical quality (Plexiglass^®^) was used for the fabrication of the POF and plain samples. Rods with a diameter of 15 mm and sheets with a depth of 1 mm were obtained from Evonik (Essen, Germany).

### 3.1. Fiber Probe Fabrication

#### 3.1.1. POF Fabrication

The POF employed in this work was fabricated in our facilities. We annealed the Plexiglass^®^ extrusion rod for 7 days in an oven with low humidity. Afterwards, we drew it directly to a 500 μm diameter only-core fiber using our POF drawing tower. This fabrication method allows us to fabricate directly only-core POFs. This way, we have complete control on the fiber diameter and the core surface roughness is much lower in comparison to the results obtained with other methods, such as the stripping of a commercial fiber.

**Figure 4:**
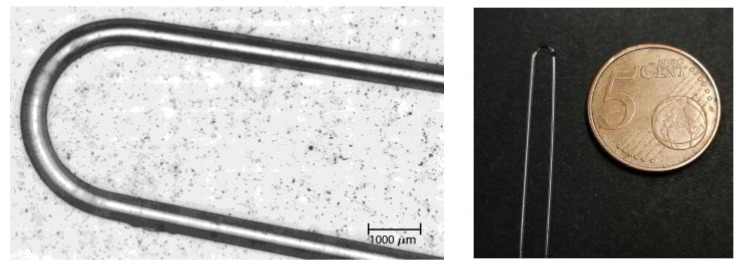
Pictures of the sensor-probe: microscopic picture (**left**) and a comparison with a 5 cent Euro coin (**right**).

After fabricating the fiber, we bent the fiber taking the following procedure: first of all, 30 cm of fiber was cut. Then, using a 2.5 mm wide glass tube as a guide, a hot-air gun set at 120 °C was directed to the section of the fiber subjected to bending, and the U-shape was carefully formed. After that, the fiber probes were washed in isopropanol for 1 h and dried in a vacuum chamber at 60 °C overnight in order to remove internal stresses. Finally, both ends of the fiber were carefully polished. The resultant sensor-probe is shown in Figure 4. 

#### 3.1.2. Surface Functionalization and ARS Aggregation

**Figure 5:**
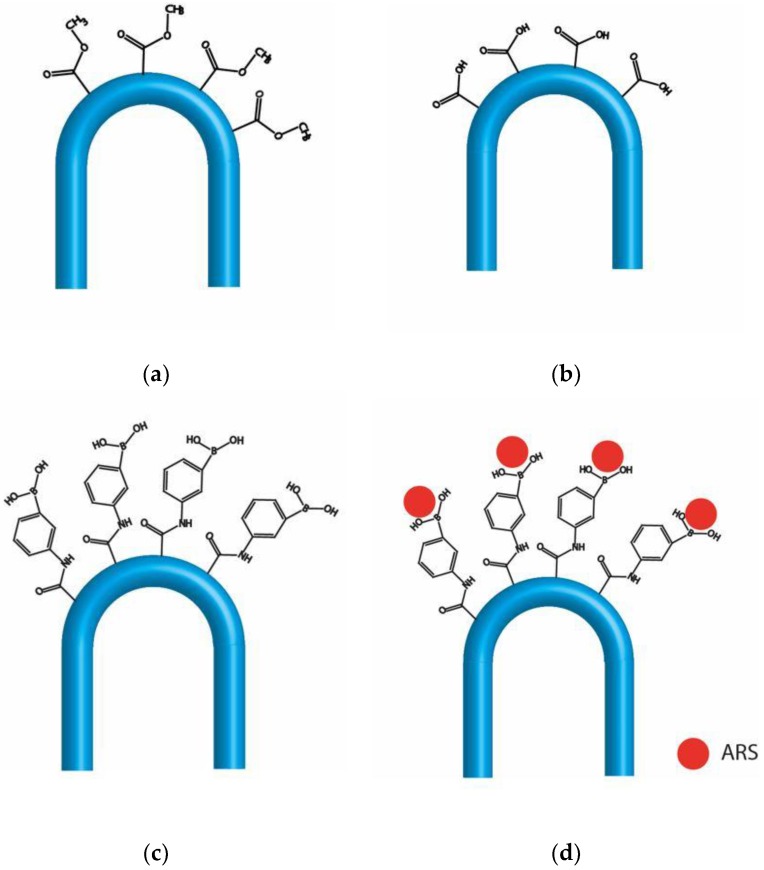
Functionalization process of the probe: (**a**) initial poly(methyl methacrylate) (PMMA) probe; (**b**) hydrolyzed probe; (**c**) PBA functionalized probe; (**d**) ARS charged probe.

The functionalization of the PMMA fiber surface was carried out by slightly modifying the method described by Fortin and Klok [24]. Briefly, starting with the U-shaped probe of Figure 5a, 2 cm of the probe were hydrolyzed by immersing them in a 3 M sulphuric acid solution in deionized water at 60 °C for 15 min, then they were washed in deionized water and left in a vacuum chamber overnight (see Figure 5b). Afterwards, an activation process of the carboxylic groups was started by immersing the hydrolyzed probes in a 0.1 M EDC and 0.2 M NHS in deionized water for 4 h at room temperature. Subsequently, they were rinsed in ethanol, and later they were left overnight in a solution of 16 mg·mL^−1^ APBA in a PBS buffer (pH 7.2). After 12 h, the probes were washed by rinsing in deionized water (20 min, three times) and, finally, they were dried in a vacuum chamber (see Figure 5c).

Once the fibers were functionalized with PBA groups, they were charged with ARS following the procedure described by Chen et al. [25]. Firstly, the tips were immersed for 3 h at room temperature in a 0.1 mg·mL^−1^ ARS solution prepared in a buffer of pH 7.4 with 50 mM TRIS and 44.7 mM HCl in deionized water. Secondly, they were washed with the buffer solution and dried in a vacuum chamber (Figure 5d).

### 3.2. Experimental Setup

**Figure 6:**
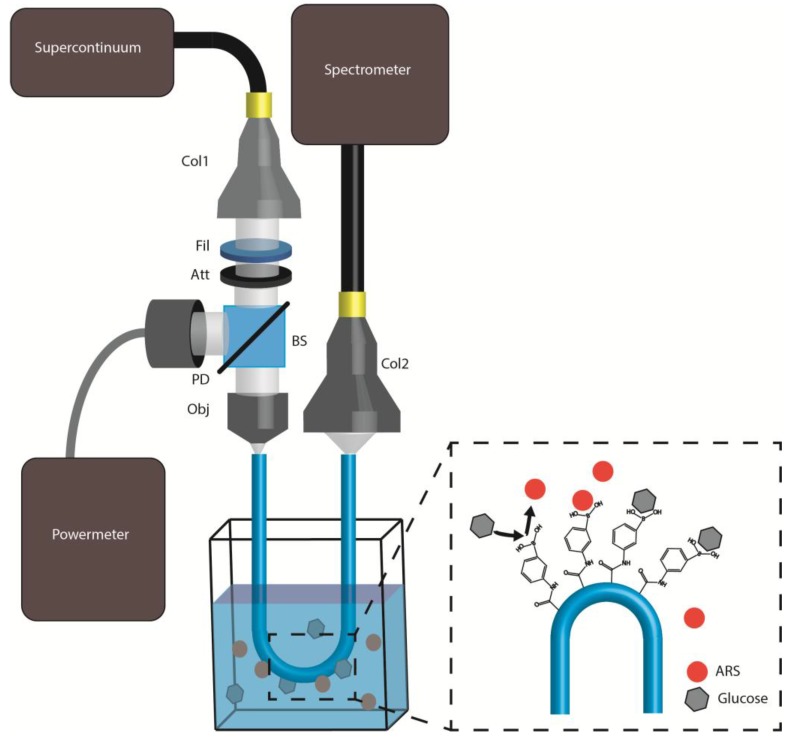
Experimental setup employed to carry out the measurements. Legend: Col1/Col2: collimators, Fil: band-pass filter (390–750 nm), Att: optical attenuator of optical density 3, BS: beam-splitter, Obj: objective, and PD: photo detector; next to the cuvette a chemical illustration of the disaggregation of the ARS is also shown.

The experimental setup employed to carry out the measurements is shown in Figure 6 together with the chemical illustration of the disaggregation of the ARS produced by the glucose. The supercontinuum light source (EQ-99-FC LDLSTM, ENERGETIQ) was launched to the fiber probes using the minimum number of components. The light emitted from the source was first collimated with a collimating lens and then filtered with a band-pass filter (390–750 nm) to remove the UV emission, and then attenuated by an OD3 attenuator to avoid saturation in the detector. In order to cancel power fluctuations of the light source, the light power was monitored using a beam splitter and a photodiode connected to a power meter. The pump light was focused on the end face of the fiber-probe using an objective (40×, 0.65 NA). The output light from the other end face was captured and focused through another collimator to the spectrometer (USB Flame 390–750 nm, Ocean Optics^®^ Inc., Largo, FL, USA).

Light detected in the spectrometer was recorded by a custom-made LabView program capable of recording and averaging 100 consecutive measurements. The spectrometer integration time can be tuned automatically during the measurements to avoid saturation effects in the original spectra.

## 4. Results and Discussion

### 4.1. Functionalization Process

The functionalization process carried out in this work requires an exhaustive control of the surface modification at each step. This is achieved by carrying out an X-ray photoelectron spectroscopy (XPS) study in the plain samples of PMMA sheets; measurements were made using a SPECS system equipped with a Phoibos 150 1D-DLD analyzer and a monochromatic radiation source Focus 500 with an Al/Ag dual anode.

**Figure 7:**
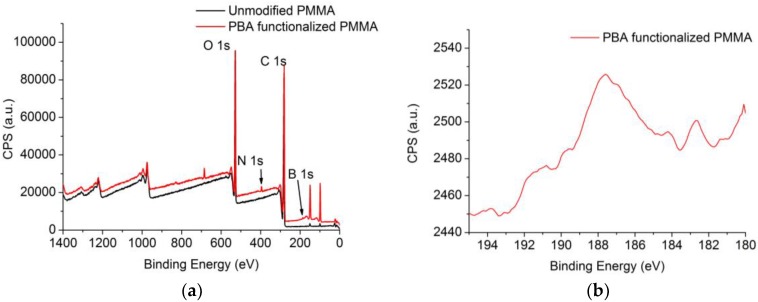
(**a**) General X-ray photoelectron spectroscopy (XPS) spectra of PBA functionalized and unmodified PMMA plain samples; (**b**) high resolution spectra of the PBA functionalized sample for boron 1s.

The XPS spectra of Figure 7a show the general spectra of unmodified PMMA and PBA-functionalized PMMA plain sheets. Regarding the specified binding energy of the boron atom (187.2 eV binding energy), a marked peak can be observed in functionalized samples. The surface functionalization with PBA groups was confirmed by high resolution spectra of the boron binding energy (Figure 7b), with 0.67% of the surface being covered by boron atoms (Table 1). 

Once the functionalization was confirmed and quantified in plain samples (Table 1), the absorption spectra of the U-shaped probes were obtained in each step of the above detailed functionalization process. As the transmitted signal power varies in different steps, all of the obtained spectra were normalized with the minimum absorption value of each measurement. 

**Figure 8:**
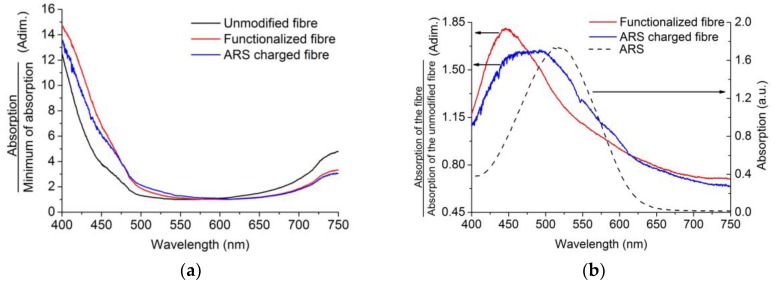
Normalized absorption curves of different probes: (**a**) unmodified, functionalized, and ARS-charged fibers; (**b**) absorption curve of functionalized and ARS-charged probe normalized with the unmodified probe and the absorption curve of ARS.

Regarding the absorption spectra shown in Figure 8a, we can observe that there is a significant difference between the unmodified fiber (black line) and the PBA-modified fiber (red line). The light entering the fiber suffers absorption caused by the PBA groups in the surface for the case of the functionalized probe, so comparing it with the unmodified probe, a strong absorption curve appears on the low wavelengths. In order to highlight the absorption caused by the functionalization process, the spectra were normalized with the unmodified probe, Figure 8b (red line). The absorption maximum at 440 nm indicates the presence of the PBA groups.

In the second step, when the U-shaped probes are charged with ARS, we observe in Figure 8b (blue line) that the ARS-charged fiber shows wider absorption caused by attached ARS with an absorption maximum at 533 nm (Figure 8b dotted line), with this absorption curve representing the combination of PBA and ARS absorptions.

### 4.2. Glucose Detection

For the detection of glucose, we recorded and compared the absorption spectra of different fibers before and after their immersion in different glucose solutions for 10 min. The probes were immersed in 6 mL of solution using polycarbonate cuvettes and only 1.5 cm of the sensitive area of the probes were immersed. Then, probes were left in air until the transmission signal was stable before spectra were recorded and, afterwards, these spectra were compared with the beginning spectra. The measurements were made using the same glucose concentration, 0.1 M, and in three different media, two of them being physiological, i.e., with a pH similar to that of the human body, 7.2–7.4. More specifically, these three media were deionized water (H_2_O), PBS buffer, and TRIS buffer. Notice that these media have pH values well above the pKa of boronic acid, as these buffers are also used for physiological media, making the interaction with the glucose possible. By testing the method in these different media, we intend to prove the suitability of the fiber probe regardless of the selected medium and its hypothetical application to a biological medium.

**Figure 9:**
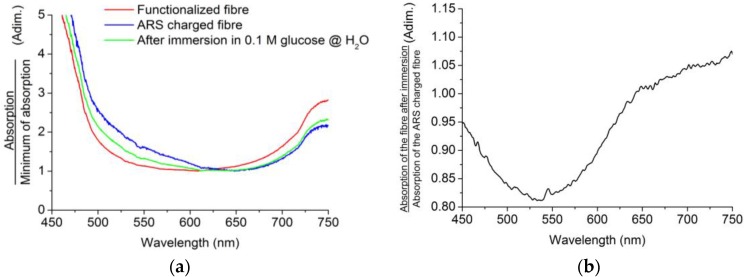
(**a**) Normalized absorption curves of the probe before and after 10 min of immersion in 0.1 M glucose solution in H_2_O; (**b**) division of ARS-charged absorption and post-immersion absorption curves.

For the H_2_O solvent, results are shown in Figure 9. From the absorption spectra (Figure 9a), it can be noticed that the absorbance of the ARS-charged fiber probe (blue line) is higher than in subsequent steps for lower wavelengths, i.e., when the same probe has been immersed in the solution with glucose and has released ARS (green line). Ideally, if the glucose were able to disrupt all the ARS-boronic bonds, we would achieve the original absorption curve corresponding to the functionalized fiber (Figure 9a, red line). To see the effect clearly, the right-hand side graph plots the normalization of the immersed absorption with the ARS-charged state, showing a dramatic decrease of the absorbance around the ARS absorption wavelength.

**Figure 10:**
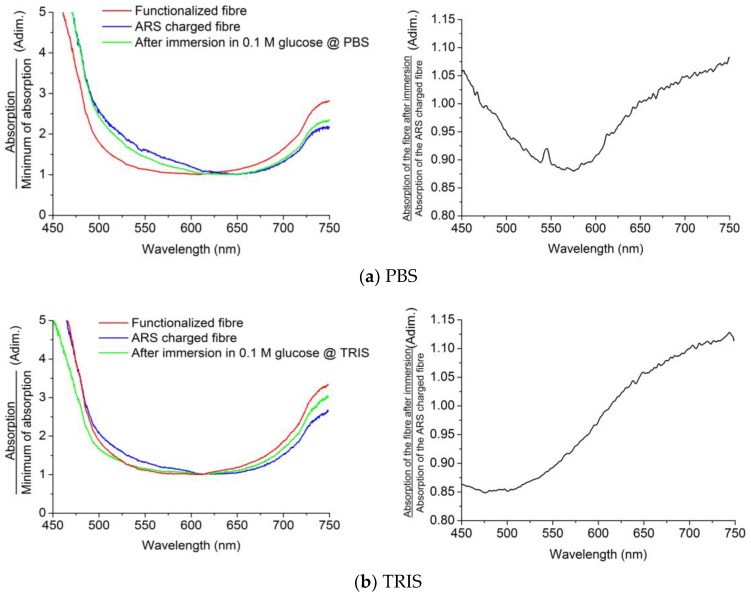
Normalized absorption curves of the probe before and after 10 min of immersion in 0.1 M glucose solution in PBS (**a**) and TRIS (**b**) (**left**); division of ARS-charged absorption and post-immersion absorption curves (**right**).

Regarding the physiological media, PBS and TRIS, their results are shown in Figure 10.

For both physiological media, the charged probe released the ARS due to the bonding of glucose on the boronic acid functionalized surface of the probe. For the case of PBS (Figure 10a), we can also observe a decrement in the absorption around 533 nm, which is highlighted in the right-hand side graph. In the case of TRIS (Figure 10b), the probe behaves qualitatively in the same way as in the other media. Indeed, it has been made sure that in the reaction no other effect might influence the absorption of the fiber probe. All in all, we can conclude that, irrespective of the media, the performance of the sensor is similar, even though the results of Figure 9 suggest a larger amount of ARS released in H_2_O.

## 5. Conclusions

In this paper, we have reported a potential glucose detection platform. The surface modification of a low-cost U-bent fiber is enough for the detection of glucose regardless of the surrounding medium, in contrast to other sensors based on evanescent wave sensing whose detection method relies on measuring the change on the refractive index. The functionalization of the PMMA with phenylboronic groups, which have high affinity to the diol groups of the glucose, allows glucose detection in physiological media. Measurement of the disaggregation of ARS in the visible range together with the simplicity of the probe paves the way for low-cost solutions for glucose detection.

## Figures and Tables

**Table 1:** Elemental composition of PMMA and PBA functionalized surface from XPS analysis.

Sample	C%	O%	N%	B%
PMMA	75.09	24.91	--	--
PBA functionalized surface	72.07	25.95	1.30	0.67
